# Describing the hidden species diversity of *Chaetozone* (Annelida, Cirratulidae) in the Norwegian Sea using morphological and molecular diagnostics

**DOI:** 10.3897/zookeys.1039.61098

**Published:** 2021-05-21

**Authors:** Maël Grosse, María Capa, Torkild Bakken

**Affiliations:** 1 University of the Balearic Island, Department of Biology, Ctra. Valldemossa km 7.5, Balearic Islands, Spain University of the Balearic Islands Palma Spain; 2 Norwegian University of Science and Technology, NTNU University Museum, Trondheim, Norway Norwegian University of Science and Technology, NTNU University Museum Trondheim Norway

**Keywords:** Continental shelf, cryptic species, integrative taxonomy, new species, North-East Atlantic, Norway, polychaetes

## Abstract

Using molecular markers and species delimitation analyses, a high diversity of bi-tentaculate Cirratulidae was discovered from the North-East Atlantic. Five new species are described: *Chaetozone pseudosetosa***sp. nov.**, *Chaetozone quinta***sp. nov.**, *Chaetozone barentsensis***sp. nov.**, *Chaetozone monteverdii***sp. nov.**, and *Chaetozone chambersae***sp. nov.** Several morphogroups are also described, even though the presence of cryptic diversity prevented naming of individual species. For each species presented, a molecular diagnostic is given from the universal barcode COI and, when available, the D1–D2 domains of the 28S rRNA. This increases the number of species in *Chaetozone* in northern European waters from ten to at least 17 species, the exact number of species remaining uncertain as taxonomic issues with older names remain unresolved.

## Introduction

Marine benthic environments in the North Sea and shelf areas in the Norwegian Sea are said to be among the best-studied areas in the world (Nygren et al. 2018). However, recent biodiversity surveys and projects aiming at mapping species occurrences, and environmental monitoring have shown that there is still more to explore and surprises to be unveiled. New species are still being discovered and described (Kongsrud et al. 2011; Nygren and Pleijel 2011; Bakken et al. 2014; Nygren et al. 2018; Capa et al. 2019), and studies of faunal characteristics (Oug et al. 2017) and distribution patterns (Eilertsen et al. 2018) show novelties not previously reported.

Polychaete worms belonging to Cirratulidae Ryckholt, 1851 are common in a diversity of marine substrates and can reach high densities, as high as up to 10.000 specimens per m^2^ in quantitative samples (Hily 1987). Therefore, they are of ecological importance and among the frequently encountered organisms in environmental monitoring. However, they show few intra-specific morphological differences and, because of this, they are known to be a taxonomic challenge and difficult to identify (e.g., Blake 1996, 2018).

The genus *Chaetozone* Malmgren, 1867, according to the latest diagnosis, is characterised, among other features, by having prominent acicular spines in noto- and neuropodia that in posterior segments arise in fascicles from elevated podial lobes or membranes that in some species almost encircle the posterior segments (Blake 2019). In Europe, a total of ten *Chaetozone* species have been reported: *C. setosa* Malmgren, 1867; *C. caputesocis* (Saint-Joseph, 1894); *C. carpenteri* McIntosh, 1911; *C. zetlandica* McIntosh, 1911; *C. corona* Berkeley & Berkeley, 1941; *C. vivipara* Christie, 1984; *C. gibber* Woodham & Chambers, 1994; *C. christiei* Chambers, 2000; *C. jubata* Chambers & Woodham, 2003; *C. elakata* Blake & Lavesque, 2017. However, the identity and generic position of some of these species (e.g., *C. caputesocis*, *C. zetlandica* and *C. vivipara*) need to be assessed, as descriptions are vague (Petersen 1999; Blake and Lavesque 2017; Le Garrec et al. 2017).

**Figure 1.**
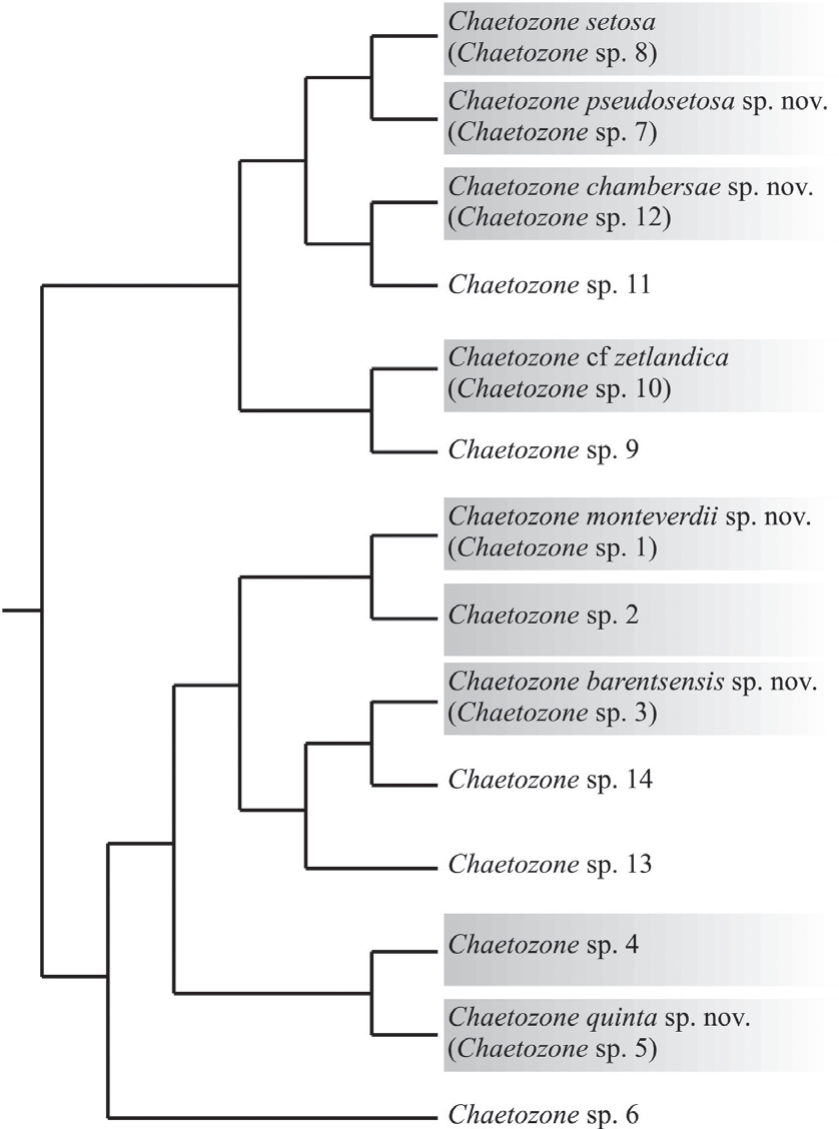
Cladogram of the different species present in Norway, after Grosse et al. (2020). The species treated in this paper are highlighted in grey.

In biodiversity assessments and monitoring surveys, Norwegian *Chaetozone* specimens are mainly sorted into four lots, named *C. setosa*, *C. jubata*, *C. christiei*, and *C. zetlandica*. But a recent study, aiming to address the species diversity of bi-tentaculate cirratulids, combining morphological examination and species delimitation analyses of DNA sequence data of the North-East Atlantic showed that total species richness had been overlooked (Grosse et al. 2020). A total of 14 *Chaetozone* species (Fig. 1), understood to be separately evolving metapopulation lineages (De Queiroz 2007), was recovered (Grosse et al. 2020). Three different scenarios for these recovered species based on DNA sequence data were identified: 1) species that unequivocally matched the diagnosis of a nominal species (like the case of *Chaetozone setosa*); 2) species that matched the diagnosis of a species complex (e.g., *C. zetlandica*); 3) species that did not match any of the currently available *Chaetozone* species or species complex diagnoses, suggesting they are undescribed.

We provide a molecular diagnosis for each species/species complex/morphogroup as several of the species presented are, at least for now, only distinguishable through DNA analyses. Molecular diagnostic characters allow the description of cryptic species in the absence of observable morphological diagnostic characters (Churchill et al. 2014; Delić et al. 2017; Teixeira et al. 2020). This is important when we know that many species discovered through molecular species delimitation analyses remain undescribed and thus unavailable for further studies (Pante et al. 2015).

In this study, we describe five new species: *Chaetozone pseudosetosa* sp. nov., *Chaetozone quinta* sp. nov., *Chaetozone barentsensis* sp. nov., *Chaetozone monteverdii* sp. nov. and *Chaetozone chambersae* sp. nov. We also describe three morphogroups, containing cryptic species and/or that cannot be linked to an existing name or described as new species as yet.

## Material and methods

### Material examined, study area and morphological observations

Nearly 100 specimens were examined in detail, and morphological comparisons were made between them and with descriptions in the literature. Most specimens have DNA vouchers previously assigned to species via DNA analyses (Grosse et al. 2020). The molecular species assignments (Fig. 1) were used as a fixed reference to differentiate between intra- and inter-specific variations in morphological characters. Most specimens were stained with Shirlastain A solution (SDL International LTD), and some of them with methylene blue. Selected specimens were examined by SEM at the University of Bergen, Norway. For morphological observations, specimens were studied with stereo and compound microscopes. Not all species recovered by Grosse et al. (2020) are formally described here, as for some species not enough specimens were available (and/or in good enough condition) to produce a description.

Specimens used in this study are deposited in the collections of the University Museum of Bergen, University of Bergen (**ZMBN**) and NTNU University Museum (**NTNU-VM**) (Bakken et al. 2020). Specimens are mainly from cruises and surveys covering areas in the Barents Sea, the Norwegian coast and shelf, the North Sea, and the Skagerrak. Several of these surveys are from projects studying polychaete diversity such as POLYSKAG (polychaetes in coastal waters of Skagerrak), BIOSKAG 2 (deep environments of the Skagerrak), PolyNor (polychaetes in the Norwegian Sea), PolyPort (polychaetes in Norwegian harbours), UNIS 2007 and UNIS 2015 (University Center in Svalbard) cruises, and monitoring surveys along the Norwegian coastline. Type specimens were made available by the Swedish Natural History Museum (**SMNH**), the National Museums of Scotland (**NMSZ**), and the British Museum (Natural History, **BMNH**).

### Molecular information

The datasets were the same as the complete *Chaetozone* COI and 28S datasets of Grosse et al. (2020). The list of specimens with GenBank accession numbers are available in Suppl. material 1. Alignments of COI and 28S sequences are available in Fasta format as Suppl. materials 2, 3 respectively. Acquisition of DNA sequences was as follows: several parapodia, a few branchiae or the posterior segments were removed for DNA extraction from 306 specimens. Tissue samples from 95 specimens were sent to the Canadian Center for DNA Barcoding, Biodiversity Institute of Ontario, University of Guelph, Guelph, Ontario, for sequencing forward and reverse strands with the primer pairs polyLCO/polyHCO or ZplankF1_t1/ZplankR1_t1 (Table 1). Tissue samples from another 211 specimens were processed at the NTNU University Museum as follows. Tissues were placed into 50 µL of QuickExtract (Epicentre) and heated at 65 °C for 60 minutes followed by 3 minutes at 95 °C in a thermo-shaker at 300 rpm. These DNA extractions were diluted in 200 µL of elution buffer (10 mM Tris-Cl, pH 8.5). Amplification of the target DNA fragments was done by Polymerase Chain Reaction (PCR). PCR mixtures contained 1.4 µL of DNA template, 0.30 µL of each primer, and 10 µL of RedTaq 1.1× MasterMix 2.0 mM MgCl2 (VWR) for a final reaction volume of 12 µL. The different pairs of primers used (jgLCO1490/jgHCO2198, CirrCOIF/CirrCOIR, or polyLCO/polyHCO for COI; and 28SC1’/28SD2 for 28S) and the PCR thermal cycling profiles are shown in Table 1. 1.5 µL of each PCR product was run for 45 minutes on a 1% agarose gel electrophoresis containing SYBR safe (Invitrogen) for DNA detection and visualised using GeneSnap from SynGene software (Version 6.08, Cambridge, UK). PCR products providing neat bands of expected size were purified with illustra ExoProStar 1-Step (GE Healthcare, Litlle Chalfont, UK). Cycle sequencing was performed on both strands by Eurofins Genomics DNA Sequencing Department (Ebersberg, Germany). Forward and reverse reads were merged into consensus sequences using Geneious 11.0.5 (https://www.geneious.com).

**Table 1.** PCR Primers: The different primer pairs used to amplify the markers used in this study and their respective cycles.

Region	Name	Length	Source	Sequence 5’-3’		Cycle
COI	jgLCO1490	~650 bp	(Geller et al. 2013)	TITCIACIAAYCAYAARGAYATTGG	34x	3 min 96 °C
	jgHCO2198		(Geller et al. 2013)	TAIACYTCIGGRTGICCRAARAAYCA		60 s 95 °C
						60 s 48 °C
						60 s 72 °C
						6 min 72 °C
	CirrCO1F	~650 bp	(Weidhase et al. 2016)	TTTTTCTACTAACCATAAAGACATTG	34x	60 s 96 °C
	CirrCO1R		(Weidhase et al. 2016)	CCGAGGAAGTGTTGAGGGA		60 s 94 °C
						60 s 53 °C
						60 s 72 °C
						5 min 72 °C
	polyLCO	~650 bp	(Carr et al. 2011)	GAYTATWTTCAACAAATCATAAAG	5x	60 s 96 °C
	polyHCO		(Carr et al. 2011)	TAMACTTCWGGGTGACCAAARAATCA		40 s 95 °C
						40 s 46 °C
						60 s 72 °C
					35x	40 s 94 °C
						40 s 51 °C
						60 s 72 °C
						7 min 72 °C
	ZplankF1_t1	~650 bp	(Prosser et al. 2013)	tTCTASWAATCATAARGATATTG	29x	60 s 95 °C
	ZplankR1_t1		(Prosser et al. 2013)	TTCAGGRTGRCCRAARAATCA		40 s 94 °C
						40 s 51 °C
						60 s 72 °C
						5 min 72 °C
28S	28SC1	D1-D2	(Le et al. 1993)	ACCCGCTGAATTTAAGCAT	29x	60 s 96 °C
	28SD2	~750 bp	(Le et al. 1993)	TCCGTGTTTCAAGACGG		30 s 95 °C
						60 s 62 °C
						60 s 72 °C
						7 min 72 °C

COI sequences were aligned using MUSCLE (Edgar 2004) implemented in Aliview 1.25 (Larsson 2014). 28S D1-D2 sequences were aligned with MAFFT 7 online version (Katoh et al. 2017) with the algorithm Q-INS-i, that considers the secondary structure of RNA, using the 200PAM/k=2 scoring matrix and a gap penalty of 1.53. The COI and 28S alignments are available in Suppl. materials 2, 3 respectively. COI p-distances were calculated using MEGA 10.0.5 (Kumar et al. 2018).

The online version of DeSignate (Hütter et al. 2020) was used to find diagnostic molecular characters for each species. Diagnostic characters for a species are defined as positions in the alignments where the nucleotides of this species are uniform, but different from the rest of the species in the alignment (Davis and Nixon 1992; DeSalle et al. 2005). DeSignate is able to find two types of diagnostic characters: single positions, or a duo of positions that are diagnostic as a combination but not individually. The positions in this duo, called combined characters by the software’s authors, can be separated by a number of other positions. We chose to select only combined characters made by adjacent positions, which are in effect a short sequence of two bases. Therefore, a k-window of 2 was used for both datasets. For each species, the positions given for diagnostic characters are that of the alignments given in Supplementary Material 2 (COI) and 3 (28S).

## Systematic account

### Genus Chaetozone

Taxon classificationAnimaliaTerebellidaCirratulidae

Malmgren, 1867

BAE1FAB1-2DFC-50D2-9384-903890EED08D

Chaetozone Malmgren, 1867: 96; Chambers 2000: 589–591; Blake 2015: 504–507; Blake 2018: 69; Blake 2019: 170–171.

#### Type species.

*Chaetozone setosa* Malmgren, 1867 by monotypy.

#### Diagnosis

**(emended).** Prostomium blunt to conical, peristomium short to elongate, usually lacking eyespots, with a pair of small nuchal slits or depressions at posterior edge; with a single pair of grooved dorsal tentacles arising from posterior edge of peristomium, or sometimes more posterior on an achaetous anterior segment, or rarely an anterior chaetiger. First pair of branchiae arising from an achaetous segment or chaetiger 1; or sometimes with first two pairs of branchiae on a single anterior segment. Branchiae laterally ciliated in distal half. Body usually expanded anteriorly, rarely with middle or posterior body segments beaded or moniliform; narrowing posteriorly or posterior end often expanded. Chaetae include capillaries on most chaetigers and sigmoid acicular spines in neuropodia and notopodia; capillary chaetae typically smooth or with sparse to dense fibrillation, fibrils generally homogeneously spread or grouped on one side of the blade, rarely arranged in concentric rings; some species with long, natatory-like capillaries, sometimes limited to gravid individuals; spines typically concentrated in posterior segments, forming distinct cinctures with spines carried on elevated membranes; cinctures with few to many spines sometimes encircling entire individual posterior segments, accompanied with none to many alternating capillaries; bidentate spines sometimes present in juveniles or occasionally accompanying unidentate spines in ventral most position of far posterior chaetigers of adults. Pygidium a simple lobe, disk-like, or with long, terminal cirrus.

#### Remarks.

**Figure 2.**
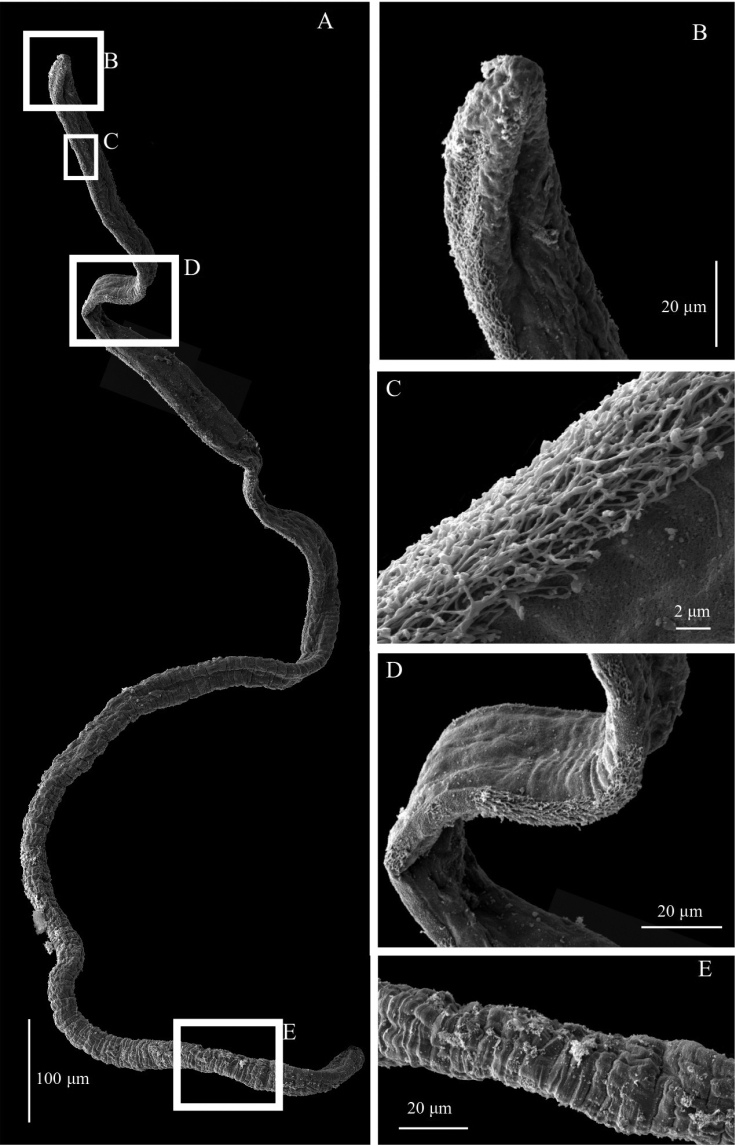
SEM of branchial anatomy, specimen ZMBN125776 (*Chaetozone* sp. 4) **A** whole branchia **B** detail of distal end **C** detail of lateral cilia **D** detail of distal half morphology **E** detail of proximal half morphology.

Based on observations from SEM images from several species in this study, the presence of cilia on the branchiae (Fig. 2) is added to the previously emended diagnosis (Blake 2019).

### Chaetozone setosa

Taxon classificationAnimaliaTerebellidaCirratulidae

Malmgren, 1867

D33B2D5F-F85F-5DB2-9BB1-272283380889

Figures 3 , 4

Chaetozone setosa Malmgren, 1867: 96, Pl. 14, fig. 84 (in part); Petersen 1999: 111; Chambers 2000: 589–591, fig. 1 (in part); Blake 2015: 504–507, figs 1, 2.
Chaetozone sp. 8 Grosse et al. 2020: fig. 4.

#### Type locality.

Isfjord, Svalbard, Norway, 55 m depth.

#### Material examined.

***Lectotype*:** Svalbard • 1 ind.; Isfjord; 06 Jun. 1864; 55 m; SMNH 1493-03. ***Paralectotypes***: Svalbard • 172 ind.; same data as for holotype; SMNH 1493-04–175. **Other material examined.** Svalbard • 7 ind.; 78.14872°N, 13.12559°E; 13 May 2015; 243 m; ZMBN125766–125769, 125837–125838, 129637; • 2 ind.; 79.55130°N, 11.22970°E; 30 Aug. 2007; 91 m; ZMBN125815–125816 • 2 ind.; 78.32855°N, 15.14712°E; 07 May 2015; 266 m; ZMBN125811, ZMBN125813 • 2 ind.; 79.70829°N, 18.17362°E; 10 May 2015; 407 m; ZMBN125817–125818 • 1 ind.; 79.68089°N, 11.13989°E; 09 May 2015; 180 m; ZMBN125770 • 1 ind.; 79.58854°N, 18.63483°E; 10 May 2015; 242 m; ZMBN125812. – Barents Sea • 1 ind.; 71.61528°N, 32.99719°E; 9 Aug. 2013; 305 m; ZMBN125764.

#### Diagnosis.

**Figure 3.**
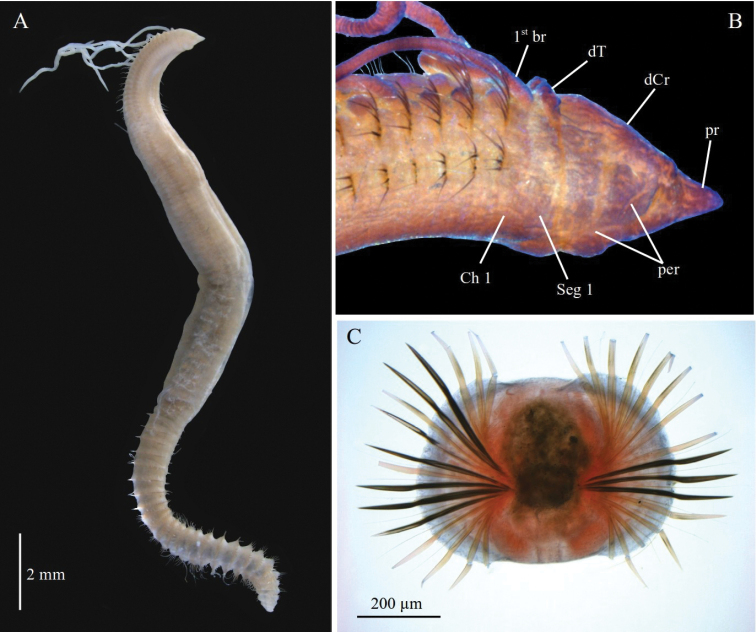
*Chaetozone setosa***A** lectotype SMNH 1493-03, in lateral view **B** paralectotype SMNH 1493-04–175, anterior end in lateral view, stained with Shirlastain A **C** paralectotype SMNH 1493-04–175, cross section of modified posterior segments. Abbreviations: br, branchiae; Ch, chaetiger; dCr, dorsal crest; dT, dorsal tentacle; per, peristomium; pr, prostomium; Seg, segment.

**Figure 4.**
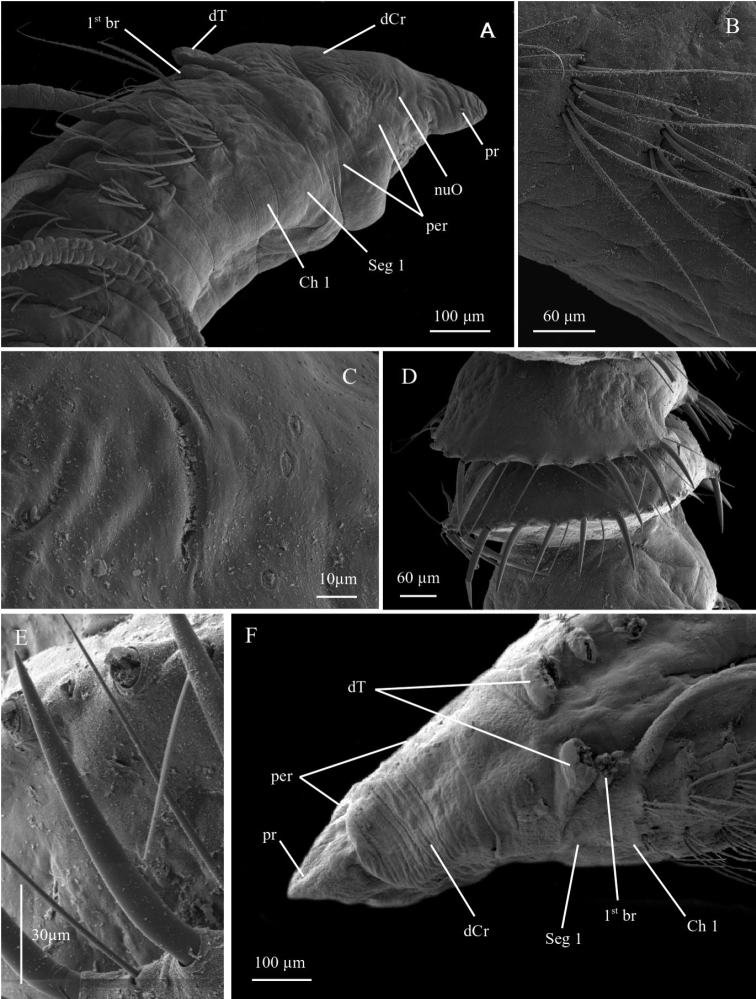
*Chaetozone setosa***A** ZMBN125817, SEM of anterior end in lateral view **B** ZMBN125768, SEM of anterior neuropodia in lateral view **C** ZMBN125817, SEM of nuchal organ, specimen **D** SEM of posterior cinctures in lateral view, specimen ZMBN125817 **E** ZMBN125817, SEM of neuropodial spine **F** ZMBN129637, SEM of anterior end in dorsal view. Abbreviations: br, branchiae; Ch, chaetiger; dCr, dorsal crest; dT, dorsal tentacle; per, peristomium; pr, prostomium; Seg, segment.

Peristomium with two large distinct annulations and dorsal crest; dorsal tentacles on posterior margin of peristomium; first pair of branchiae on distinct segment 1 (achaetous); posterior segments developed in full cinctures with up to 20–26 spines per parapodia (Figs 3, 4) (based on Blake 2015).

#### Molecular diagnosis.

COI: 220: G. 28S: 545–546: AC (based on 36 COI sequences and 19 28S sequences).

#### Distribution.

Barents Sea, Svalbard, White Sea, ~ 80–400 m depth.

#### Remarks.

A lectotype (Fig. 3A) and 172 paralectotypes from Svalbard were designated by Petersen (1999), from which a thorough redescription and illustrations were provided by Blake (2015). As many early described polychaete species, *C. setosa* has been reported from all around the world (Chambers 2000; Oug et al. 2014), before being restricted to the Arctic and subarctic areas in northern Europe (Blake 2015), which corresponds to the area covered by Malmgren’s type series: Spitsbergen (Svalbard), Finnmark (northern Norway) and Bohüslan (western Sweden). Specimens were recently collected from the type locality in Svalbard, as well as other areas along Northern Europe, including from the localities given in Malmgren. Genetic analyses revealed the presence of two distinct, yet morphologically identical species (Fig. 1): one present in Svalbard, the Barents Sea, and the White Sea (Fig. 1; *Chaetozone* sp. 8), and the other present in the Norwegian Sea, the North Sea, and the Skagerrak (Fig. 1; *Chaetozone* sp. 7) (Grosse et al. 2020). The former species included specimens collected at the type locality of *C. setosa*, consequently interpreted as members of the type species of *Chaetozone*. The other one is described herein and named *C. pseudosetosa* sp. nov. This species is morphologically identical to *Chaetozone pseudosetosa* sp. nov., described below. Methylene blue stains the peristomium with transversal bands of varying intensity, as well as anterior segments and posterior cinctures also with transversal bands, mostly ventrally (Blake 2015). *Chaetozone pseudosetosa* sp. nov. shows a similar pattern. Genetic distance in the COI marker between *Chaetozone setosa* and other congeners in the area mostly ranges from 20% to 25%, except for a minimum of 8% divergence with *C. pseudosetosa* sp. nov. (Table 2).

**Table 2.** COI p-distances between and within Norwegian *Chaetozone* species. The number of base differences per site from averaging over all sequence pairs within each group are shown. Species described and discussed in this paper are denoted with an “°”.

	*Chaetozone monteverdii* sp. nov.º		*Chaetozone barentsensis*º		*Chaetozone quinta*º	*Chaetozone pseudosetosa* sp. nov.º	*Chaetozone setosa*º		*Chaetozone cf zetlandica*º		*Chaetozone chambersae* sp. nov.º		
	(*Chaetozone* sp. 1)	*Chaetozone* sp. 2º	(*Chaetozone* sp. 3)	*Chaetozone* sp. 4º	(*Chaetozone* sp. 5)	(*Chaetozone* sp. 7)	(*Chaetozone* sp. 8)	*Chaetozone* sp. 9º	(*Chaetozone* sp. 10)	*Chaetozone* sp. 11	(*Chaetozone* sp. 12)	*Chaetozone* sp. 13	*Chaetozone* sp. 14
*Chaetozone monteverdii* sp. nov.º	0.0019	0.2372	0.2470	0.2677	0.2532	0.2572	0.2452	0.2400	0.2350	0.2268	0.2194	0.2343	0.2557
(*Chaetozone* sp. 1)													
*Chaetozone* sp. 2º	0.2372	0.0048	0.2627	0.2674	0.2840	0.2718	0.2625	0.2667	0.2669	0.2466	0.2447	0.2444	0.2737
*Chaetozone barentsensis*º	0.2470	0.2627	0	0.2356	0.2364	0.2490	0.2328	0.2559	0.2508	0.2459	0.2389	0.1587	0.0953
(*Chaetozone* sp. 3)"													
*Chaetozone* sp. 4º	0.2677	0.2674	0.2356	0.0015	0.0950	0.2705	0.2594	0.2472	0.2405	0.2457	0.2410	0.2496	0.2353
*Chaetozone quinta*º	0.2532	0.2840	0.2364	0.0950	0	0.2553	0.2620	0.2458	0.2314	0.2568	0.2585	0.2508	0.2379
(*Chaetozone* sp. 5)													
*Chaetozone pseudosetosa* sp. nov.º	0.2572	0.2718	0.2490	0.2705	0.2553	0.011	0.0889	0.2090	0.2079	0.1737	0.1889	0.2299	0.2556
(*Chaetozone* sp. 7)													
*Chaetozone setosa*º	0.2452	0.2625	0.2328	0.2594	0.2620	0.0889	0.004	0.2045	0.2041	0.1701	0.1949	0.2214	0.2369
(*Chaetozone* sp. 8)													
*Chaetozone* sp. 9	0.2400	0.2667	0.2559	0.2472	0.2458	0.2090	0.2045	0.0033	0.1069	0.1931	0.1988	0.2486	0.2553
Chaetozone cf zetlandicaº	0.2350	0.2669	0.2508	0.2405	0.2314	0.2079	0.2041	0.1069	0.0014	0.1994	0.1994	0.2492	0.2581
(*Chaetozone* sp. 10)													
*Chaetozone* sp. 11	0.2268	0.2466	0.2459	0.2457	0.2568	0.1737	0.1701	0.1931	0.1994	0.0061	0.1088	0.2219	0.2594
*Chaetozone chambersae* sp. nov.º	0.2194	0.2447	0.2389	0.2410	0.2585	0.1889	0.1949	0.1988	0.1994	0.1088	0.0053	0.2225	0.2463
(*Chaetozone* sp. 12)													
*Chaetozone* sp. 13	0.2343	0.2444	0.1587	0.2496	0.2508	0.2299	0.2214	0.2486	0.2492	0.2219	0.2225	0.0008	0.1694
*Chaetozone* sp. 14	0.2557	0.2737	0.0953	0.2353	0.2379	0.2556	0.2369	0.2553	0.2581	0.2594	0.2463	0.1694	0.0102

The number of base differences per site from averaging over all sequence pairs within each group are shown. Species described and discussed in this paper are denoted with an “º”.

### Chaetozone pseudosetosa sp. nov.

Taxon classificationAnimaliaTerebellidaCirratulidae

244692DA-BEAE-5B02-9436-49E89C490E06

http://zoobank.org/8CAA7808-CB69-4983-874D-19A5FF982EFF

Figures 5 , 6

Chaetozone sp. 7 Grosse et al. 2020: fig. 4.

#### Type locality.

Drøbak, Oslofjorden, south of Storskjær, Norway, 31 m depth.

#### Material examined.

***Holotype*:** Oslofjorden, Norway • 59.6562°N, 10.6081°E; 20 Oct. 2014; 31 m; ZMBN125756. ***Paratypes***: Oslofjorden, Norway • 3 ind.; 59.6444°N, 10.6192°E; 21 Oct. 2014; 106 m; NTNU- VM74516–74518 • 1 ind.; 59.05485°N, 10.250467°E; 29 May 2011; 70 m; NTNU-VM74514 • 1 ind.; 59.89017°N, 10.75551°E; 20 Sep. 2018; 12 m; NTNU-VM76534 • 1 ind.; 59.89731°N, 10.73703°E; 20 Sep. 2018; 8 m; NTNU-VM76547. – North Sea • 1 ind.; 59.28789°N, 5.32506°E; 08 Jun. 2014; 76 m; ZMBN125790 • 1 ind.; 59.02985°N, 5.44881°E; 10 Jun. 2014; 59 m; ZMBN125789 • 3 ind.; 60.269686°N, 5.197750°E; 26 Jul. 2014; 120 m; NTNU-VM74525–74526, 74528 • 1 ind.; 59.76022°N, 5.49682°E; 08 Jun. 2014; 40 m; ZMBN125787 • 1 ind.; 60.90389°N, 7.16813°E; 17 Nov. 2012; 115 m; ZMBN125795 • 1 ind.; 58.24753°N, 6.53673°E; 03 Feb. 2016; 155 m; ZMBN125824 • 1 ind.; 60.60332°N, 5.09513°E; 6 Mar. 2017; 94 m; ZMBN125780. – Norwegian Sea • 1 ind.; 63.44753°N, 10.62730°E; 07 Feb. 2018; 77 m; NTNU-VM74602 • 1 ind.; 63.437891°N, 10.50624°E; 04 Sep. 2018; 4 m; NTNU-VM75900. – Barents Sea • 1 ind.; 70.262°N, 31.083833°E; 16 Apr. 2014; 126 m; NTNU-VM74499. – Sweden • 2 ind.; 58.866667°N, 11.1°E; 2005; 70 m; ZMBN129641, 129647 • 2 ind.; 58.8°N, 11.1°E; Nov. 2018; 60 m; ZMBN129643, ZMBN129644.

#### Diagnosis.

**Figure 5.**
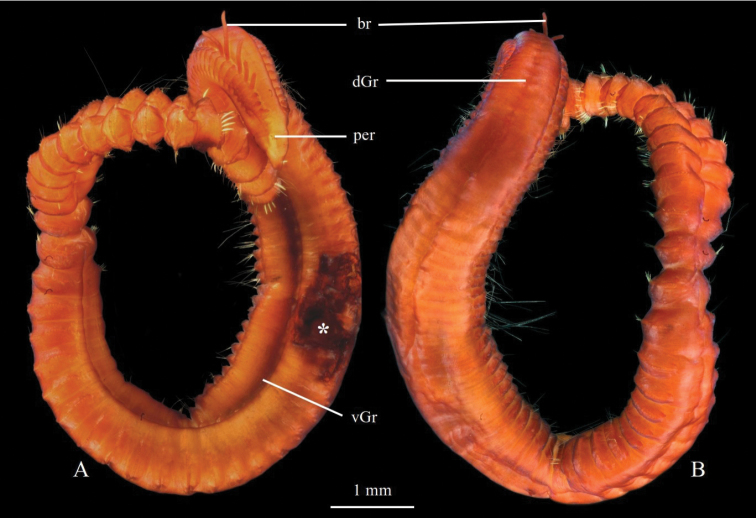
*Chaetozone pseudosetosa* sp. nov., holotype ZMBN125756 **A** holotype in ventral view, stained with Shirlastain A **B** holotype in dorsal view, stained with Shirlastain A. Abbreviations: br, branchiae; dGr, dorsal groove; per, peristomium; vGr, ventral groove; a star (*) indicates where parapodia were removed for DNA analyses.

**Figure 6.**
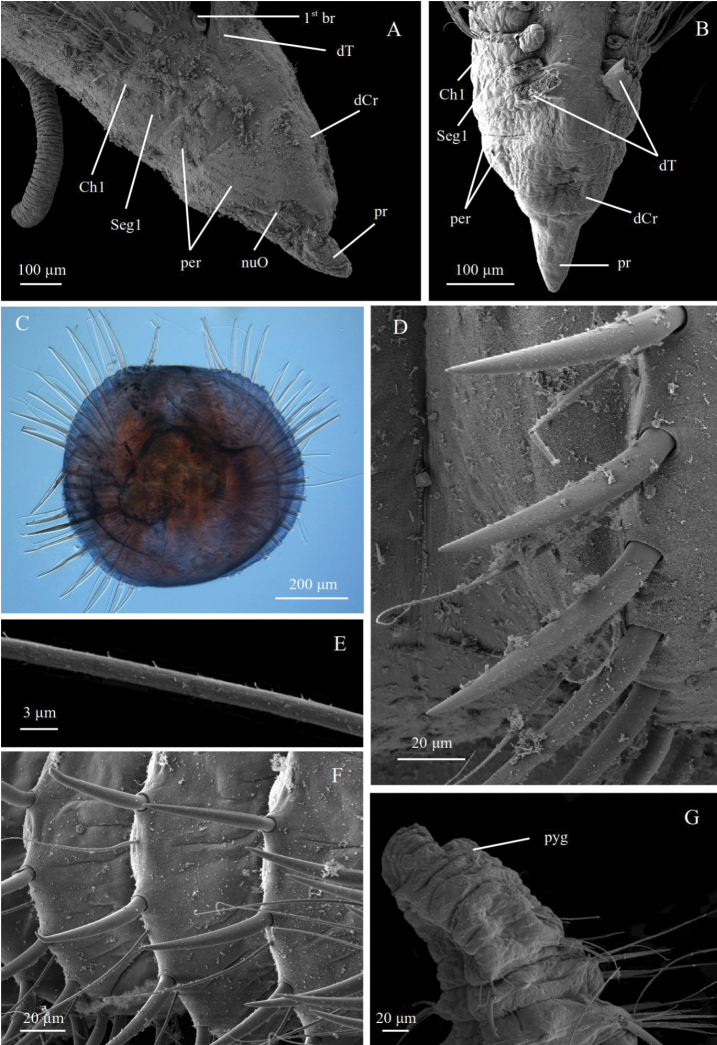
*Chaetozone pseudosetosa* sp. nov. **A** paratype ZMBN125780, SEM of anterior end in lateral view **B** paratype ZMBN129644, SEM of anterior end in dorsal view **C** paratype NTNU-VM74602, cross section of posterior segment **D** paratype ZMBN125780, SEM neuropodial spines **E** paratype ZMBN129644, SEM of notopodial capillary **F** Paratype ZMBN129644, SEM of posterior neuropodia **G** paratype ZMBN129643, SEM of pygidium. Abbreviations: br, branchiae; Ch, chaetiger; dCr, dorsal crest; dT, dorsal tentacle; nuO, nuchal organ; per, peristomium; pr, prostomium; pyg, pygidium; Seg, segment.

Peristomium with two large distinct annulations and dorsal crest; paired tentacles on posterior margin of peristomium; first branchiae on distinct segment 1 (achaetous); posterior segments developed in full cinctures with up to 20–26 spines per parapodia (Figs 5, 6).

#### Molecular diagnosis.

COI: 223: C; 471–472: CA; 349–350: TT (based on 45 COI sequences).

#### Description.

A medium to large species, holotype incomplete, with 78 segments (70–106), 16.5 mm long (12–20 mm), up to 1.2 mm wide (Fig. 5). Colour in ethanol white to light tan. Body elongate, wider in midbody segments, narrowing anteriorly and posteriorly; circular to oval in cross section. Anterior first 15–20 segments 5–6 × wider than long, progressively lengthening 3 × longer posteriorly (Fig. 5). Thin, shallow dorsal groove from segment 10–15. Distinct ventral groove along most of body (Fig. 5).

Prostomium short, long as two third of peristomium, conical to triangular, tapering to rounded anterior tip, without annulations; eyespots absent; nuchal organs as narrow slits at posterior margin of prostomium (Fig. 6A, B). Peristomium as long as wide, with two large rings of similar length, distinct laterally, weakly distinct or invisible dorsally on dorsal crest; dorsal crest little to well developed, covering all peristomium, slightly overlapping prostomium anteriorly, extending posteriorly between dorsal tentacles, up to anterior margin of chaetiger 1 (Fig. 6A, B). Dorsal tentacles arising from posterior margin of peristomium, well separated (Fig. 6A, B). First pair of branchiae arising from segment 1 (achaetous), posterior to dorsal tentacles (Fig. 6A, B). Second pair of branchiae arising from chaetiger 1, dorsally and slightly posterior to notopodia. Subsequent branchiae similarly placed. Branchiae or branchial scars on most chaetigers until development of cinctures.

Parapodia as low mounds or ridges in anterior and middle segments, progressively developing into high, elevated membranes and into complete cinctures from segment 63 (50–85) (Figs 5, 6C, F). 6–11 short capillaries per neuro- and notopodia throughout, smooth; 2–4 long natatory-like capillaries per notopodia from segment 20 or 21 to 70–72, up to 1.5 × longer than body width, smooth (Fig. 6E). 8–13 spines per neuro- and notopodia, from segment 42–54 in neuropodia and segment 46–54 in notopodia, unidentate, sigmoid, rarely slightly crossing dorsally in posterior cinctures (Fig. 6C, D, F). Alternating capillaries usually between all spines, of similar length of longer than spines.

Pygidium with terminal anus and with small rounded ventral lobe (Fig. 6G).

#### Methylene blue staining pattern.

A moderately distinctive pattern. Methyl blue stains the peristomium lightly in transversal bands. Dorsum does not stain. Transversal lines of dark blue dots are present along anterior segments, more or less dense but never strong, not covering the whole length of the segments, creating a light stripe pattern anteriorly. Rows of dark blue dots also present on most mid and posterior parapodia, not conspicuous and only lateral in midbody segments.

#### Etymology.

This species is named *pseudosetosa* because it looks identical to *C. setosa* and has been identified as such until now.

#### Remarks.

Although this species is morphologically indistinguishable from *C. setosa*, it is a distinct species, and thus we felt it was important to name it. Cryptic species are important to take into account (Nygren 2014), which is difficult to do if they are not named, do not have a type, and are not diagnosed either morphologically or molecularly. In the absence of diagnostic morphological characters to distinguish it from *C. setosa*, molecular diagnostic characters can be of help (Nygren and Pleijel 2011; Parapar et al. 2020). In particular, *C. setosa* is known as a bioindicator and distinguishing between *C. setosa* and *C. pseudosetosa* sp. nov. can be important in that regard.

*Chaetozone pseudosetosa* sp. nov. COI distance with other species in the area mostly ranges from 20% to 25%, with a minimum of 8% with *Chaetozone setosa* (Table 2).

#### Distribution.

Norwegian coast and shelf, Skagerrak, North Sea, 4–160 m depth. One specimen is recorded from Finnmark, which means it may be sympatric with *C. setosa* in this area.

### Chaetozone quinta sp. nov.

Taxon classificationAnimaliaTerebellidaCirratulidae

C8794CCF-A41E-5F8B-9996-E02D39383B47

http://zoobank.org/12A6B992-A58C-42C5-9F71-5F6FCC3DD350

Figure 7

Chaetozone sp. 5 Grosse et al. 2020: fig. 4.

#### Type locality.

Søra Kjerringasundet, east of Sotra, Bergen, 75 m depth.

#### Material examined.

***Holotype*:** North Sea • 1 ind.; 60.32652°N, 5.14085°E; 02 Sep. 2014; 75 m; ZMBN125802. ***Paratypes***: North Sea • 1 ind.; 61.04889°N, 4.9723°E; 15 Jul. 2015; 161 m; ZMBN125777 • 1 ind.; 60.50728°N, 5.00028°E; 30 Nov. 2015; 66 m; ZMBN125807. **Other material examined.** North Sea • 3 ind.; 60.355133°N, 5.168967°E; 10 Apr. 2018; 92 m; ZMBN138610–138612.

#### Diagnosis.

**Figure 7.**
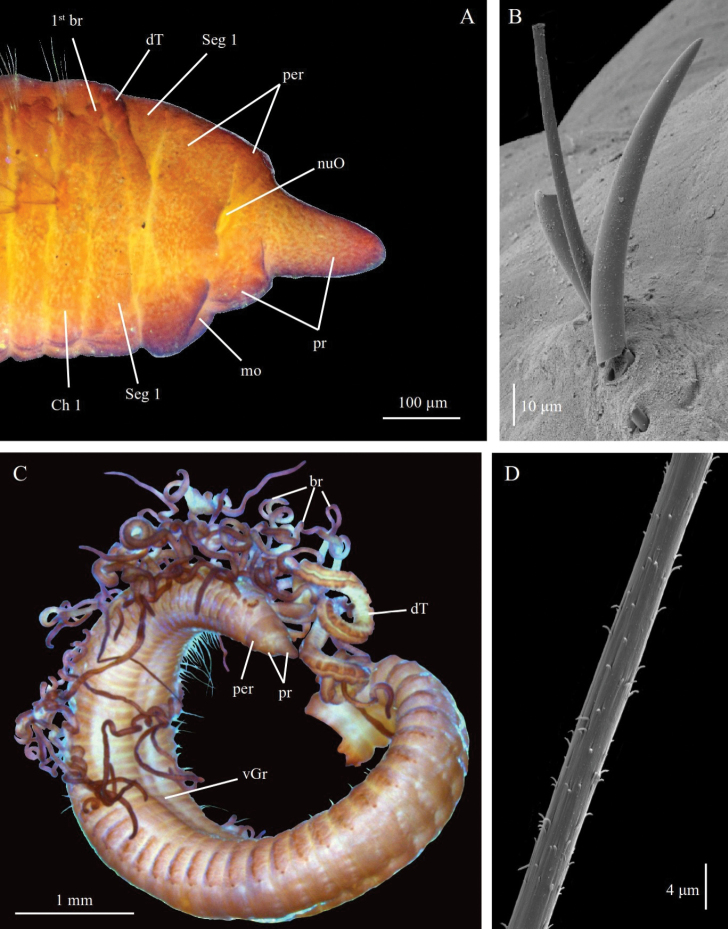
*Chaetozone quinta* sp. nov. **A** ZMBN138610, anterior end in lateral view, stained with Shirlastain A **B** paratype ZMBN125807, SEM of neuropodial spine **C** holotype ZMBN125802 in lateral view, stained with Shirlastain A **D** paratype ZMBN125807, SEM of capillary chaetae. Abbreviations: br, branchiae; Ch, chaetiger; dT, dorsal tentacles; mo, mouth; nuO, nuchal organ; per, peristomium; pr, prostomium; Seg, segment; vGr, ventral groove.

Prostomium ventrally bi-annulated; peristomium short and without annulations; wide ventral groove; paired tentacles and first branchiae on segment 1 (achaetous); posterior segments developed in incomplete cinctures bearing 9–11 spines per parapodia (Fig. 7).

#### Molecular diagnosis.

COI: 446: T; 475: G; 539: T; 548: T; 607: G; 349–350: GG; 361–362: AG; 486–487: TA (based on 2 COI sequences).

#### Description.

A medium species, holotype incomplete, 61–70 segments, 6–8 mm long, 0.6–1 mm wide. Colour of ethanol preserved specimens white to light tan. Body elongate, narrowing progressively anteriorly, 2–3 × wider at anterior third than at anterior end. Round in cross section anteriorly, widening progressively to a flatten oval at anterior third. Anterior segments 5 × higher and wider than long. Midbody segments 10 × wider and 5 × higher than long. Posterior segments 2.5 × higher and 3 × wider than long. Thin shallow dorsal groove, best visible anteriorly. Wide shallow ventral groove along entire body (Fig. 7C).

Prostomium longer than peristomium, conical, tapering to rounded anterior tip; with ventrally and laterally distinct posterior annulation above mouth, as long as segment 1; eyespots absent; nuchal organs simple slits at posterior margin of prostomium, above posterior annulation (Fig. 7A). Peristomium short, as long as segment 1 ventrally, as long as two segments dorsally, without annulations, overlapping with prostomium anteriorly (Fig. 7A). Dorsal tentacles arising from segment 1 (achaetous), well separated (Fig. 7A). First pair of branchiae arising from segment 1, immediately behind tentacles (Fig. 7A). Segment 1 longer than chaetiger 1, achaetous, weekly bi-annulated (Fig. 7A). Second pair of branchiae arising from chaetiger 1, dorsal and slightly posterior to parapodia (Fig. 5A). Subsequent branchiae similarly placed. Branchiae or branchial scars present on most chaetigers until development of cinctures.

Parapodia as low mounds or ridges in anterior and middle segments, developing into relatively low incomplete cinctures from segment 45–50, arising on each side but not completing over venter and dorsum. Five or six short capillaries in neuropodia throughout, in notopodia from development of spines, smooth; 5–7 medium capillaries in notopodia, from chaetigers 1–29, twice as long as neuropodial capillaries, smooth; one or two long capillaries in notopodia from segment 12 to 27–29, smooth (Fig. 7D). Five or six spines per neuropodia from segment 27–29, four or five spines per notopodia from segments 31 or 32, unidentate, short, rather spread out (Fig. 7B). Alternating capillaries between most spines except ventralmost, slightly longer than spines.

Pygidium with terminal anus and with a short, rounded ventral lobe.

#### Methylene blue staining pattern.

Prostomium except tip, peristomium and sides of segment 1 retain a dark blue stain, while rest of body does not stain.

#### Etymology.

*Quinta* is the cardinal adjective for fifth in the feminine nominative singular, as this species has always been “*Chaetozone* sp. 5”. It is also named with a thought for a friend and colleague who is named after the same number.

#### Remarks.

This species is easily distinguished from other species of *Chaetozone* in Norwegian waters by its distinct bi-annulated prostomium, short peristomium, and segment 1 (achaetous) bearing both tentacles and first branchiae. For other species of *Chaetozone* in the area, prostomium is always simple. The methylene blue staining pattern is also unique and easily recognisable, with most of prostomium except the distal tip, peristomium and sides of segment 1 retaining a dark blue pattern, unlike the rest of the body.

*Chaetozone quinta* sp. nov. COI distance with other species in the area mostly ranges from 23% to 28%, with a minimum of 9% with *Chaetozone* sp. 4 (Table 2).

#### Distribution.

Norwegian coast and shelf, ~ 60–160 m depth.

### Chaetozone barentsensis sp. nov.

Taxon classificationAnimaliaTerebellidaCirratulidae

5F22DFE1-A62C-5671-BAE3-2C2FA6EDD0A2

http://zoobank.org/5A4E27EE-59D3-48E9-9F89-2C061FCDDDDF

Figure 8

Chaetozone sp. 3 Grosse et al. 2020: fig. 4.

#### Type locality.

Barents Sea, 337 m depth.

#### Material examined.

***Holotype*:** Barents Sea • 1 ind.; 71.056°N, 29.655667°E; 21 Apr. 2014; 337 m; NTNU-VM74492. ***Paratypes***: Barents Sea • 7 ind.; 71.056°N, 29.655667°E; 21 Apr. 2014; 337 m; NTNU-VM74493–74498, ZMBN129638 • 2 ind.; 71.187833°N, 28.943167°E; 23 Apr. 2014; 380 m; NTNU-VM74489, ZMBN129639. – North Sea • 1 ind.; 60.173°N, 5.003°E; 22 Apr. 2011; 6 m; ZMBN95707.

#### Diagnosis.

**Figure 8.**
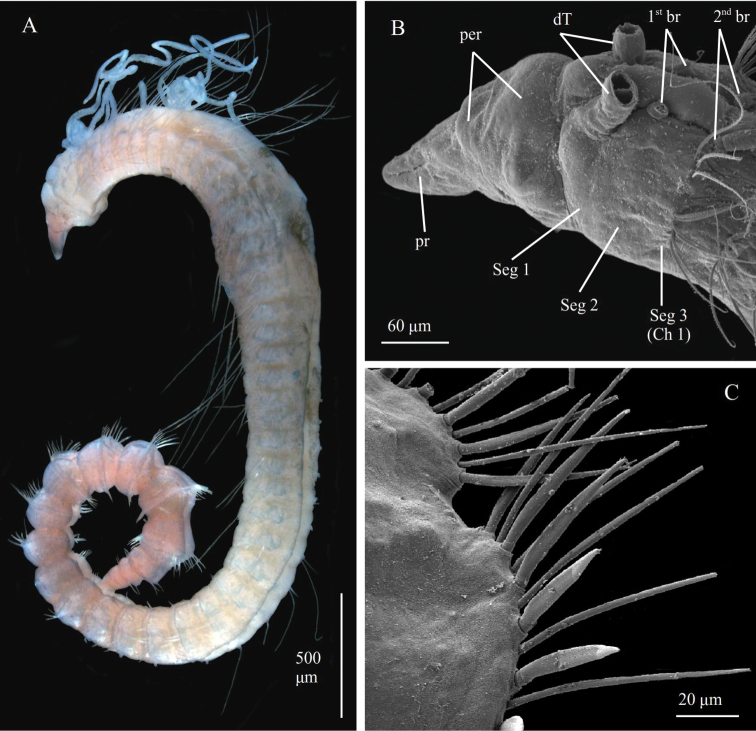
*Chaetozone barentsensis* sp. nov. **A** holotype NTNU-VM74492 in lateral view, stained with Shirlastain A **B** paratype ZMBN129638, SEM of anterior end in lateral view **C** paratype ZMBN129639, SEM of posterior notopodia. Abbreviations: br, branchiae; Ch, chaetiger; dT, dorsal tentacles; per, peristomium; pr, prostomium; Seg, segment.

Dorsal tentacles on distinct segment 1 (achaetous); first branchiae on indistinct segment 2 (achaetous); approximately 22 short, flat spines per parapodia in posterior segments (Fig. 8).

#### Molecular diagnosis.

COI: 158: A; 214: G; 518: A; 283–284: CT; 289–290: TA. 28S: 37: A; 419: T; 457: A; 461: A; 462: A; 510: C (based on 13 COI sequences and 3 28S sequences).

#### Description.

A small species, holotype complete, 40–48 segments, 4–5 mm long, 0.25 mm wide (Fig. 8A). Colour in ethanol white to light grey. Body elongate, without any distinct enlargement, rather round in cross section, slightly flattened in posterior half. Anterior segments 4 × higher than long. Posterior segments 2 × higher than long. Dorsal groove over posterior half. Ventral ridge along entire body.

Prostomium as long as peristomium, 2 × longer than high, conical, without annulations; eyespots absent; nuchal organs simple slits at posterior margin of prostomium (Fig. 8B). Peristomium short, as long as two anterior segments, sometimes appears partially divided in two annulations, one anterior to mouth and one bearing mouth (Fig. 8B). Dorsal tentacle arising from segment 1 (achaetous), well separated. Segment 1 achaetous, distinct from peristomium (Fig. 8B). First pair of branchiae arising from segment 2 (achaetous), aligned with dorsal tentacles and second pair of branchiae (Fig. 8B). Segment 2 achaetous, often indistinct from segment 1 and chaetiger 1 (Fig. 8B). Second pair of branchiae arising from chaetiger 1, dorsal to notopodia (Fig. 8B). Subsequent branchiae similarly placed. Branchiae or branchial scars present on most chaetigers until development of cinctures.

Parapodia as low mounds or ridges in anterior and middle parts, progressively developing into elevated membrane and into complete cinctures around segment 25–37 (Fig. 8A). Thirteen smooth short capillary chaetae present in all chaetigers 2–4 long chaetae in anterior notopodia, up to several times body width. 22 spines from segment 24–28 in neuropodia and segment 25–29 in notopodia, short, slightly curved, dorsalmost spines thin and rounded in cross section, gradually flattening and widening towards most lateral positions (Fig. 8C). Alternating capillaries present between all spines, longer than spines (Fig. 6C).

Pygidium with terminal anus and with a small rounded ventral lobe.

#### Methylene blue staining pattern.

No particular pattern. Prostomium and peristomium retains slightly more stain than rest of body.

#### Etymology.

The name comes from the Barents Sea, where the species was found.

#### Remarks.

*Chaetozone barentsensis* sp. nov. is similar in general appearance to *C. setosa*, which is found in the same area, although it is smaller (up to 5 mm vs. 3 cm for *C. setosa*) and differs, in particular, in the position of its tentacles which arise from the first achaetous segment vs. posterior margin of the peristomium for *C. setosa*, the presence of a ventral ridge instead of a groove, and the shape of its spines which are significantly shorter than that of *C. setosa*.

*Chaetozone barentsensis* sp. nov. COI distance with other species in the area mostly ranges from 23% to 26%, with a minimum of 9% with *Chaetozone* sp. 14 (Table 2).

#### Distribution.

*Chaetozone barentsensis* sp. nov. is found in the Barents Sea, ~ 400 m depth. One specimen was found on the Norwegian coast outside Bergen at 6 m depth.

### Chaetozone monteverdii sp. nov.

Taxon classificationAnimaliaTerebellidaCirratulidae

0CD9D597-AF9D-5A7F-A134-E18EC4BBFADF

http://zoobank.org/0B70368F-1A73-41DA-8A98-DCA3F2ACAC24

Figures 9 , 10

Chaetozone sp. 1 Grosse et al. 2020: fig. 4.

#### Type locality.

Norwegian Sea, north-west of Bergen, 280 m depth.

#### Material examined.

***Holotype*:** Norwegian Sea • 1 ind.; 61.37705°N, 2.11215°E; 31 May 2014; 280 m; ZMBN98250. ***Paratypes***: North Sea • 1 ind.; 59.56729°N, 5.21568°E; 26 Apr. 2017; 328 m; ZMBN125786 • 1 ind.; 62.35117°N, 6.16178°E; 21 Jul. 2012; 243 m; ZMBN125783 • 1 ind.; 60.2593°N, 5.13703°E; 13 Jun. 2017; 248 m; ZMBN116562 • 2 ind.; 59.99°N, 5.35°E; 27 Jun. 2007; 250 m; NTNU-VM74506, ZMBN129648.

#### Comparative material.

*Chaetozone jubata*: Paratypes: Faroe-Shetland channel • 2 ind.; 61.5.57°N, 2.4093°W; Jul. 1996; 710 m; NMSZ.1999.237.4–5.

#### Diagnosis.

**Figure 9.**
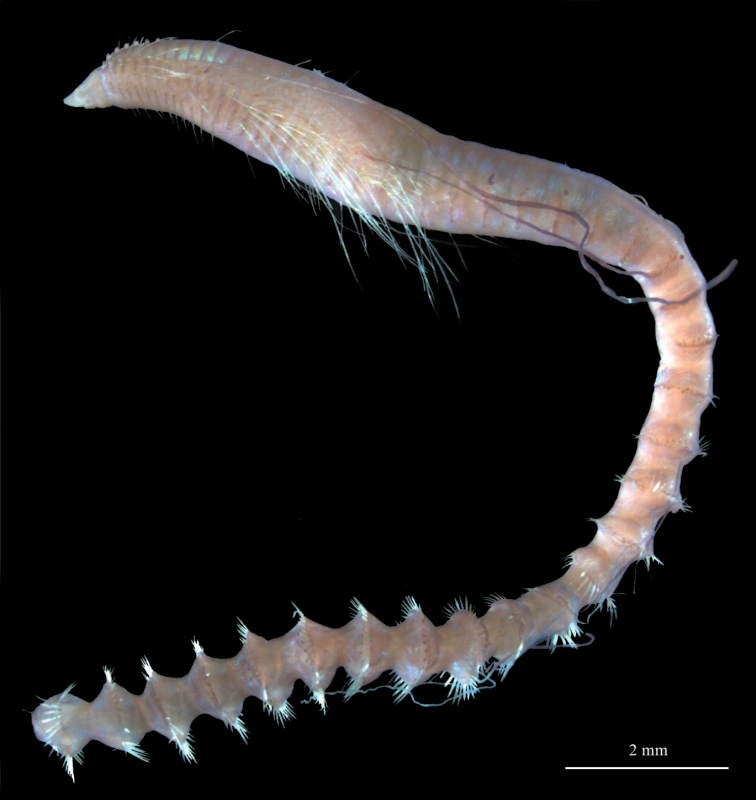
*Chaetozone monteverdii* sp. nov., Holotype ZMBN98250 in lateral view.

**Figure 10.**
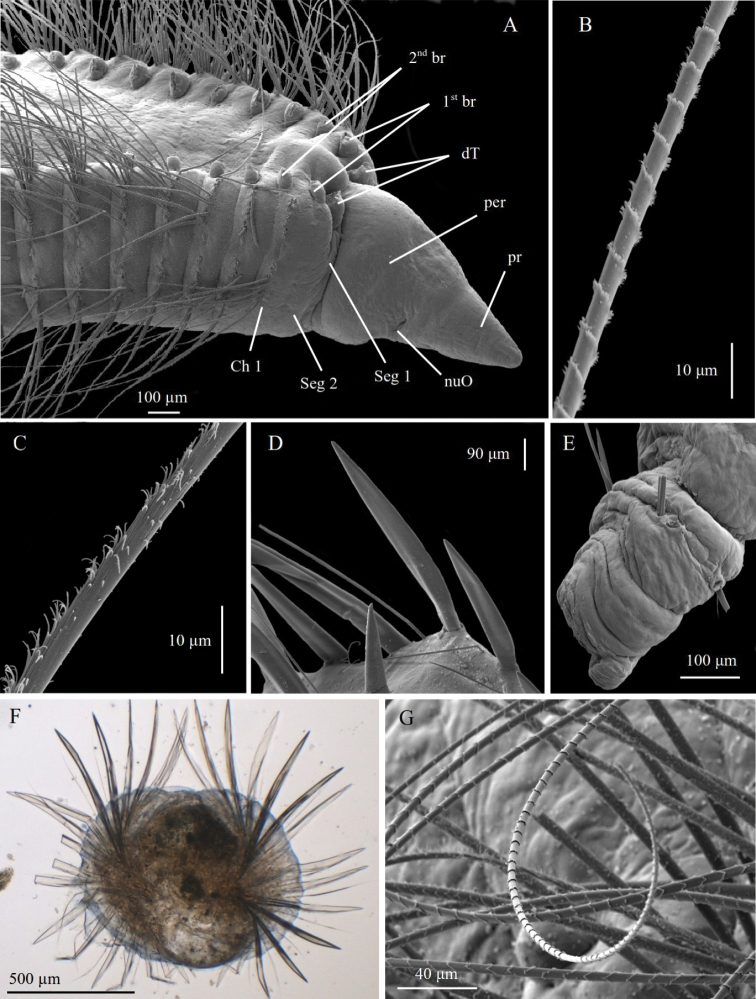
*Chaetozone monteverdii* sp. nov. **A** paratype ZMBN125786, SEM of anterior end in lateral view **B** paratype ZMBN125786, SEM of long segmented notopodial capillary **C** paratype ZMBN125786, SEM of smooth notopodial capillary **D** paratype ZMBN129648, SEM of notopodial spine **E** paratype ZMBN129648, SEM of pygidium in lateral view **F** paratype ZMBN116562, cross section of posterior segment **G** paratype ZMBN129648, SEM of long segmented notopodial capillary. Abbreviations: br, branchiae; Ch, chaetiger; dT, dorsal tentacles; nuO, nuchal organ; per, peristomium; pr, prostomium; Seg, segment.

Prostomium fused with peristomium, giving the anterior end a drop-like appearance; dorsal tentacles on segment 1 (achaetous), first pair of branchiae on segment 2 (achaetous); ventral ridge; long capillary chaetae on expanded anterior with fibrils arranged in distinctive transversal rows, numerous, long, broad and flat spines on high complete cinctures (Figs 9, 10).

#### Molecular diagnosis.

COI: 97: G; 110: C; 145: C; 199: G; 232: 277: G; C; 281: G; 282:T; 356: C; 363: T, 459: T; 485: G, 515: A; 530: T; 564–565: CC, 37–38: TA. 28S: 58: A; 69: T; 440: A; 416–417: CT; 453–454: CT; 454–455: TG; 460–461: GC (based on ten COI sequences and 13 28S sequences).

#### Description.

A large species, holotype incomplete, 56 segments (51–56), 20 mm long (14–25 mm), 1.5 mm wide. (Fig. 9). Body elongate, larger anteriorly, narrowing towards the anterior end and in posterior half, oval to flattened oval in cross section anteriorly, round in cross section posteriorly. Anterior segments narrow and crowded, 5 or 6 × higher and wider than long, lengthening and enlarging progressively after first 10–15 segments to 2 × higher than long in posterior segments. Thin, shallow dorsal groove over first 10–15 segments. Prominent ventral ridge along anterior half of body.

Prostomium as long as peristomium, conical, blunt, fused with peristomium, without annulations; eyespots absent; nuchal organs simple slits on posterior margin of prostomium (Fig. 10A). Peristomium short, as long as three segments, without annulations, overlapping segment 1 posteriorly, in large specimens much narrower than first segments giving whole head a characteristic “drop shape” clearly set off from rest of body (Fig. 10A). Dorsal tentacles on segment 1 (achaetous), well separated (Fig. 10A). Segment 1 achaetous, not completing dorsally, in large specimens, wider than peristomium but not as wide as segment 2 (achaetous) which can cover it on the side so that it is only “facing forward” (Fig. 10A). First pair of branchiae arising from segment 2 (achaetous) (Fig. 10A). Small dorsal crest over segments 1–4 in some specimens. Second pair of branchiae arising from chaetiger 1, dorsal to notopodia (Fig. 10A). Subsequent branchiae similarly placed (Fig. 8A). Branchiae or branchial scars present on most chaetigers until development of cinctures.

Parapodia as low mounds or ridges in anterior segments, progressively developing into elevated membranes and into complete cinctures around segment 38–43, with deep constrictions between the segments (Figs 9, 10F). 7–12 short capillary chaetae in anterior notopodia, approximately 12 in anterior neuropodia, smooth basally and with thin, dense fibrils along one edge from middle (Fig. 10C). Approximately seven very long capillary chaetae in notopodia from chaetiger 3 or 4 up to chaetiger 25, segmented, each segment like a cylinder diagonally cut in cross section with thin fibrils along the edge, difficult to see with light microscopy but obvious with SEM (Fig. 10B, G). 12–16 spines per neuropodia from segment 29–32, 12–14 per notopodia from segment 29–34, long, with a broad flattened elongated leaf shaped blade, slightly folded along its length, longer in notopodia, often crossing over dorsum (Fig. 10D, F). Alternating capillary chaetae between all spines, as long or shorter than spines (Fig. 10D, F).

Pygidium with terminal anus and with a small rounded ventral lobe (Fig. 10E).

#### Etymology.

This species is named after Claudio Monteverdi, an Italian composer, author of the operatic scena ‘Il combattimento di Tancredi e Clorinda’, amongst other pieces.

#### Methylene blue staining pattern.

No strong pattern. Some dark stained dots appear after differentiation on the pygidium, the posterior side of some parapodia, and the underside of some segments.

#### Remarks.

The size and volume of the prostomium, peristomium and the first segments varies in some specimens, which do not exhibit the characteristic “drop-shaped” head and enlarged anterior segments shown on Figures 9, 10, or sometimes only slightly. The prostomium and peristomium are, however, always fused and the arrangement of the dorsal tentacles and first pairs of branchiae is always the same, with dorsal tentacles on segment 1 (achaetous) and first pair of branchiae on segment 2 (achaetous).

This species is similar to *C. jubata* in the general appearance, presence of long chaetae (several times the body width) along the anterior part of the body (from approximately the 2^nd^–4^th^ chaetigers to approximately the 25^th^ for both species), and the distinctive ample posterior cinctures with big characteristic leaf shaped spines. *Chaetozone jubata* was described as having the tentacular palps originating dorsally from the posterior margin of the third peristomial ring. On the two paratypes of *C. jubata* examined, the peristomial rings are difficult to distinguish and there seems to be either a last, short peristomial ring or an achaetous segment between the tentacular palps and chaetiger 1, on which no branchiae was found. In *Chaetozone monteverdii* sp. nov. we interpret the tentacular palps as originating from a first achaetous segment, which is very distinct from the peristomium. *Chaetozone monteverdii* sp. nov. also differs from *C. jubata* in the nature of the long chaetae (segmented in *C. monteverdii* sp. nov.), the size of the specimens (up to 8 mm reported for *C. jubata* and 20 mm for *C. monteverdii* sp. nov.), the presence of a ventral ridge (a groove in *C. jubata*) and the number of short capillary chaetae in anterior parapodia (5–10 in *C. jubata* and 19–24 in *C. monteverdii* sp. nov.). *Chaetozone monteverdii* sp. nov. is readily distinguished from most other species of *Chaetozone* in the area by the complete fusion of prostomium and peristomium and its distinctive drop-like head shape (in most specimens), the long capillary chaetae restricted to the anterior part of the body, and the amplitude of the posterior cinctures, along with the size and number of spines fully or nearly encircling them. However, it is very similar to *Chaetozone* sp. 2 and *Chaetozone* sp. 4 (in this paper), from which it is so far only distinguished by the nature of the long capillary chaetae, which present fibrils arranged in transversal rows unique to this species, and the presence of a ventral ridge instead of a ventral groove. *Chaetozone monteverdii* sp. nov., *Chaetozone* sp. 2, and *Chaetozone* sp. 4 are all found in the same geographic area (Norwegian coast and shelf) and in the same range of depths (~ 200–600 m).

*Chaetozone monteverdii* sp. nov. COI distance with other species in the area ranges from 22% to 26% (Table 2).

#### Distribution.

Norwegian coast and shelf, offshore and in the fjords, south of the Trondheimsfjord, ~ 200–300 m depth.

### Chaetozone chambersae sp. nov.

Taxon classificationAnimaliaTerebellidaCirratulidae

9660A6AD-42CA-51EB-B6A5-88DA7D5BABCE

http://zoobank.org/F973A1E3-5203-40B4-8C7B-173620CFA091

Figure 11

Chaetozone sp. 12 Grosse et al. 2020: fig. 4.

#### Material examined.

***Holotype*:** North Sea • 1 ind.; 58.274250°N, 2.644216°W; 18 Jul. 2008; 56 m; NTNU-VM74546. ***Paratypes***: North Sea • 3 ind.; 51.354333°N, 2.796667°E; 14 Sep. 2010; 22 m; NTNU-VM74486–74487, ZMBN129642 • 1 ind.; 57.777177°N, 2.905357°W; 17 Jul. 2008; 37 m; NTNU-VM74537 • 1 ind.; 51.352833°N, 2.862°E; 14 Sep. 2010; 18.9 m; NTNU-VM74483 • 1 ind.; 51.3575°N, 2.8041°E; 14 Sep. 2010; 21.5 m; NTNU-VM74485.

#### Comparative material.

*Chaetozone christiei*: Holotype: North Sea • 1 ind.; Nov. 1982; 55.32°N, 1.36°W; NMSZ.1998. 122. Paratypes: North Sea • 2 ind.; Nov. 1982; 55.32°N, -1.36°W; NMSZ.1998.123.

#### Diagnosis.

**Figure 11.**
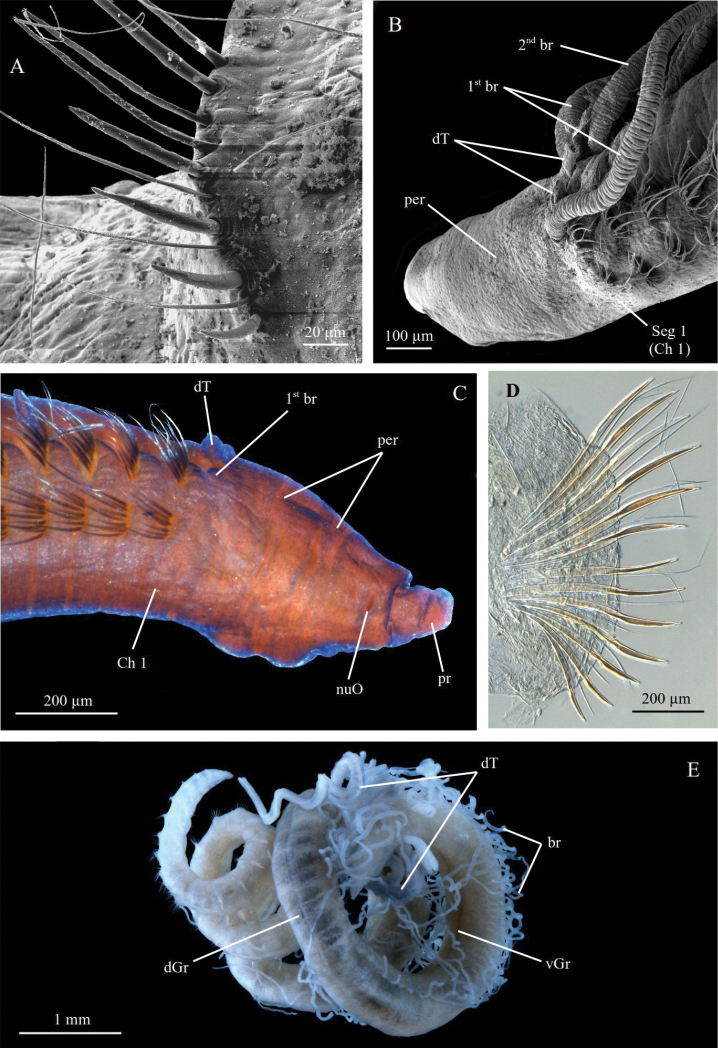
*Chaetozone chambersae* sp. nov. **A** paratype ZMBN129642, SEM of posterior neuropodia **B** paratype ZMBN129642, SEM of anterior end in dorso- lateral view **C** paratype NTNU-VM74486, anterior end in lateral view **D** paratype D NTNU-VM74487,cross section of posterior parapodia **E** holotype NTNU-VM74546 in dorso-lateral view. Abbreviations: br, branchiae; Ch, chaetiger; dT, dorsal tentacles; nuO, nuchal organ; per, peristomium; pr, prostomium; Seg, segment.

Dorsal and ventral grooves along the body; paired tentacles on the posterior margin of the peristomium; first branchiae between peristomium and first chaetiger, beside tentacles; capillary chaetae short and thick; 13–16 spines per parapodia in posterior segments (Fig. 11).

#### Molecular diagnosis.

COI: 163: C; 517: G; 512–513: GG. 28S: 89: C; 638: T (based on 17 COI sequences and 10 28S sequences).

#### Description.

A medium species, holotype complete, 124 segments (84–129), 10 mm long (7.5–14) (Fig. 11E). Colour in ethanol white to light tan. Body elongate, narrowing anteriorly and posteriorly, round in cross section, dorsum and venter rounded. Anterior and midbody segments 5–6 × wider than long. Posterior segments 1.5 × wider than long. Thin, shallow dorsal groove along most of body. Faint to distinct ventral groove along most of body.

Prostomium long like two third of peristomium, conical, blunt; eyespots absent; nuchal organs simple slits at posterior margin of prostomium. Peristomium as long as four or five segments, rarely with two distinct annulations, of approximately equal size, partially fused with chaetiger 1 posteriorly. Dorsal tentacles arising from the posterior margin of peristomium, well separated (Fig. 11B, C). First pair of branchiae arising between peristomium and chaetiger 1, just beside dorsal tentacles (Fig. 11B, C). Second pair of branchiae arising from posterior margin of chaetiger 1, dorsal to notochaetae (Fig. 11B). Subsequent chaetigers with branchiae similarly placed. Branchiae or branchial scars present on most chaetigers until development of cinctures.

Parapodia as low mounds or ridges in anterior and middle segments, progressively developing posteriorly into elevated membranes and into incomplete cinctures around segment 90, arising laterally and dorsally, not developing ventrally (Fig. 11A, D). Approximately 20 capillary chaetae per anterior parapodia, short, smooth, thick, and sometimes darkly pigmented in anterior chaetigers (Fig. 11C). Seven or eight spines per neuropodia from segment 43–60, 6–8 in spines per notopodia from segment 46–67, short, unidentate, pointy, slightly curved, transparent (Fig. 11A, D). Alternating capillaries between most spines, rarely two between two spines, thin, up to three times longer than the spines in notopodia (Fig. 11A, D).

Pygidium with terminal anus and with a small triangular ventral lobe.

#### Etymology.

This species is named after Dr Susan Chambers, for her work on European cirratulids.

#### Remarks.

This species is similar to *C. christiei* in general appearance and in having low, incomplete cinctures with short spines, although it has a few more spines per parapodia than *C. christiei*. *Chaetozone chambersae* sp. nov. differs from *C. christiei* in the position of the first pair of branchiae, which are on the posterior margin of the peristomium, beside the tentacular palps, rather than on the first chaetiger. *Chaetozone chambersae* sp. nov. differs from *C. setosa* notably in the absence of a first achaetous segment and fewer, shorter spines.

*Chaetozone chambersae* sp. nov. is found in British waters, from where many European Cirratulidae species are described. Particular care should be used when identifying cirratulids from this area because of the presence of several undescribed species (Christie 1985; Chambers 2000), the presence of variability in the type material of some species (e.g., *C. christiei*) and the revelation of a much higher diversity that expected in this group (Grosse et al. 2020). It will be important to get better understanding of the British fauna using DNA-based methods.

*Chaetozone chambersae* sp. nov. COI distance with other species in the area generally ranges from 18% to 25% with a minimum at 10% with *Chaetozone* sp. 11 (Table 2).

#### Distribution.

North Sea, northeast of Scotland, and off Belgium, from ~ 20 to 60 m depth.

### Chaetozone cf. zetlandica

Taxon classificationAnimaliaTerebellidaCirratulidae

McIntosh, 1911

32A5F47D-F825-5774-A3D9-34F1518665D8

Figures 12 , 13

Chaetozone zetlandica McIntosh, 1911: 171; Southern, 1914: 115, pls 12, 13, fig. 29A–K.
Caulleriella zetlandica : Day, 1967: 507; Woodham and Chambers 1994: 311 figs 2, 4.
Heterocirrus zetlandica : Fauvel 1927: 99, fig. 34i–n.
Chaetozone sp. 10 Grosse et al. 2020: fig. 4.

#### Material examined.

Norwegian Sea • 1 ind.; 60.54973°N, 5.22897°E; 20 Apr. 2017; 37 m; ZMBN125779 • 2 ind.; 60.17295°N, 5.00315°E; 24 Apr. 2014; 6 m; ZMBN125819–125820 • 1 ind.; 60.51035°N, 5.19158°E; 30 Nov. 2015; 32 m; ZMBN125808 • 1 ind.; 60.173°N, 5.003°E; 23 Apr. 2014; 6 m; ZMBN95386 • 1 ind.; 63.43206°N, 10.37709°E; 07 Sep. 2018; 5 m; NTNU-VM76410 • 1 ind.; 59.97547°N, 5.73998°E; 19 Sep. 2018; 10 m NTNU-VM76478 • 1 ind.; 60.3188833°N, 5.2552833°E; 12 Sep. 2019; 48 m; NTNU-VM76407 • 1 ind.; 60.39426°N, 5.30989°E; 10 Sep. 2018; 4.5 m; NTNU-VM76409.

#### Comparative material.

*Chaetozone zetlandica*: Holotype: Shetland • 1 ind.; Jul. 1867; 170 m; BMNH 1921.5.1.3232.

#### Diagnosis.

**Figure 12.**
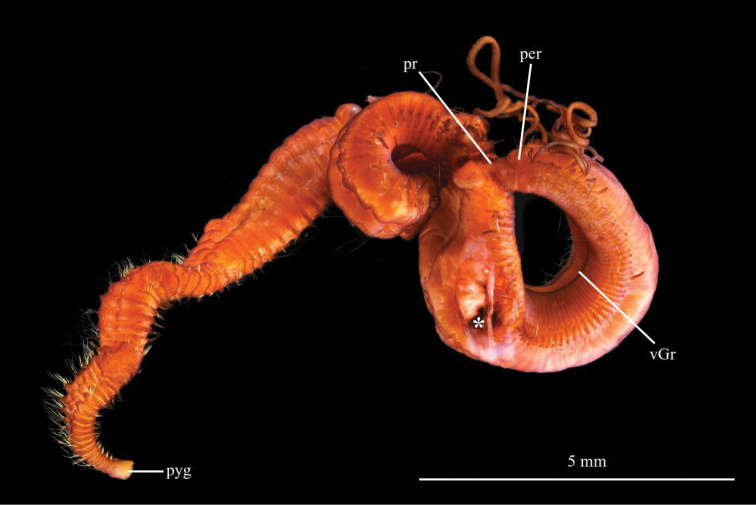
Chaetozone cf. zetlandica. NTNU-VM76410 in dorso-lateral view, stained with Shirlastain A. Abbreviations: per, peristomium; pr, prostomium; pyg, pygidium; vGr, ventral groove; an asterisk (*) indicates where the parapodia were removed for DNA analyses.

**Figure 13.**
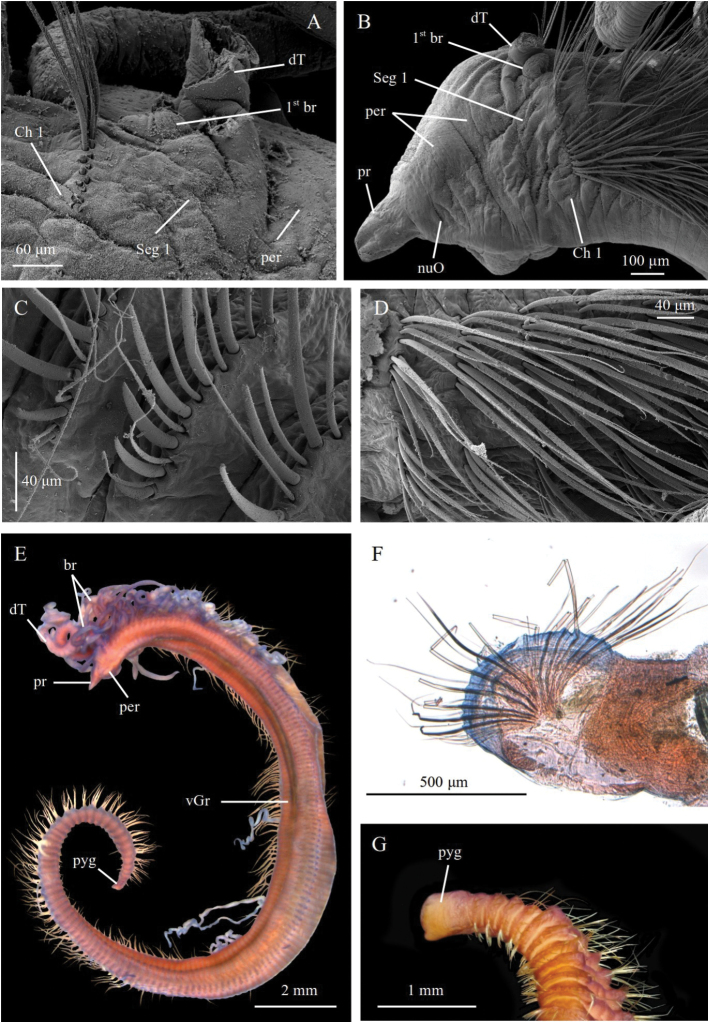
Chaetozone cf. zetlandica**A** ZMBN125808, SEM of dorsal tentacle and first branchiae **B** ZMBN125808, SEM of anterior end in lateral view **C** ZMBN125808, SEM of neuropodial posterior spines **D** ZMBN125808, SEM of anterior parapodia and capillary chaetae **E** ZMBN125820 in lateral view, stained with Shirlastain A **F** ZMBN95386, cross section of posterior parapodia **G** NTNU-VM76410, pygidium in lateral view, stained with Shirlastain A. Abbreviations: br, branchiae; Ch, chaetiger; dT, dorsal tentacles; nuO, nuchal organ; per, peristomium; pr, prostomium; Seg, segment.

All segments narrow, of approximately the same length; red eyespots; peristomium dorsally rounded; paired tentacle on incomplete segment 1 (achaetous); first branchiae on segment 1 (achaetous); posterior end flattened, posterior chaetigers with low, incomplete cinctures (Figs 12, 13).

#### Molecular diagnosis.

28S: 636: T; 675: T (based on 9 COI sequences and 4 28S sequences).

#### Description.

A large species, 130–154 segments, up to 22 mm long, 3 mm wide, 2 mm high. Body elongate, slightly widening after the middle before narrowing and flattening in posterior quarter, round–oval in cross section anteriorly. Anterior and midbody segments approximately all the same length, all very short, approximately 10 × higher than long, lengthening progressively to 6 × wider than long in posterior segments. Thin dorsal groove in midbody; large ventral groove (Figs 12, 13E).

Prostomium short, one third of peristomium, conical, blunt, without annulations; red eyespots around the nuchal organs; nuchal organs simple slits at posterior margin of prostomium (Fig. 13B). Peristomium short, long as five segments, higher than long, dorsum rounded, two annulations of approximately equal length, second one shorter ventrally and extending dorsally posteriorly between dorsal tentacles (Fig. 13B). Dorsal tentacles arising from segment 1 (achaetous), clearly separated (Fig. 13A, B). First pair of branchiae beside or directly posterior to paired tentacles, on segment 1 (achaetous) (Fig. 13A, B). Second pair of branchiae on chaetiger 1, just above notopodia. Subsequent branchiae similarly placed. Branchiae or branchial scars on all chaetigers including posterior cinctures.

Parapodia as low mounds or ridges in anterior and midbody chaetigers, progressively developing into elevated membrane and into incomplete cinctures around segment 120, encircling only the sides of posterior segments (Figs 12, 13C, E–G). 11–15 Smooth short thick capillary chaetae in neuropodia in anterior and midbody chaetigers, in notopodia along entire body, arranged in two rows in anterior segments (Fig. 13A, D). Smooth short thin chaetae in neuropodia and notopodia in midbody and posterior segments, alternating with thick capillaries in midbody segments, alternating with spines in posterior segments. 7–10 pointed acicular spines in posterior neuropodia from segment 100 (Fig. 13C, F). 7–9 long capillary tipped spines in posterior notopodia from segment 110 (Fig. 13F). Alternating capillaries between all spines (Fig. 13F).

Pygidium with terminal anus, long, cylindrical, with a short ventral lobe and dorsal mound overlapping last two chaetigers (Fig. 13G).

#### Methylene blue stain.

No particular pattern. Prostomium and peristomium stain a bit darker than rest of body, except for a band joining nuchal organs dorsally, and dorsum barely shows any stain.

#### Remarks.

The specimens examined resemble the fragment of the holotype of *Chaetozone zetlandica*. *Chaetozone zetlandica* was described from a unique posterior fragment that is in poor condition and lacks most chaetae. One neuropodium is complete and shows unidentate spines arranged in a distinct armature on an elevated membrane. Nothing could be seen from the notopodia. Southern (1914) described a number of complete specimens from Scotland he identified as *C. zetlandica* based on the original fragment from McIntosh (1911). Woodham and Chambers (1994) also examined material from Scotland that they attributed to this species but placed it in the genus *Caulleriella*. They formed this new combination because they did not see any spines in the notopodia and observed some bidentate spines in the neuropodia of small specimens. They did show, however, what they called “awl-shaped” capillary chaetae, as thick as the acicular spines, but longer, and indeed terminating into thin capillary instead of having a blunt tip. These “awl-shaped” capillary chaetae are also shown arranged in a distinct armature, with alternating capillary chaetae on an elevated membrane. As Blake (2018) states, the chaetae of *Caulleriella* are not arranged in cinctures and as the “awl-shaped” capillary chaetae of this species might indeed just be spines, *Caulleriella zetlandica* is strongly suspected to be *Chaetozone zetlandica*. Woodham and Chambers (1994) provided a detailed redescription from a quantity of specimens they also compared to the fragment holotype. Specimens examined in our study mostly confirm to their description. Where they describe the dorsal tentacles as arising from a third peristomial annulation, we describe the dorsal tentacles of our material as arising from segment 1 (achaetous), which is a difference in interpretation rather than a difference in morphology. Woodham and Chambers also describe the first pair of branchiae as arising from the first chaetiger, but their SEM pictures show that it either arise at the anterior of this chaetiger (in addition to a second pair placed posteriorly on the same chaetiger), or from the third peristomial annulation. This last interpretation would be similar to the description we make of our material. However, this possible variation in the position of branchiae cannot be confirmed until more specimens are available.

The specimens described herein are also very similar to *Chaetozone* sp. 9 from Grosse et al. (2020), a species not described here as only two specimens were available. New observations from ongoing work indicate a third genetic clade in this complex. Either of these lineages could be *C. zetlandica*. This is why the species described here as *Chaetozone* sp. 10 is referred with reservations. Although available descriptions in the literature are detailed (Southern 1914; Woodham and Chambers 1994), results presented here show the presence of putative cryptic species that at present cannot with certainty be attributed to the name McIntosh (1911) had available specimens for his original description. We suggest this is solved by examining specimens representing all genetic lineages and assigning the name *C. zetlandica* to one of them. Moreover, due to the condition of the holotype and the un-informativeness of the original description, the validity of the name *Chaetozone zetlandica* may need to be re-assessed in the future.

The COI distance between Chaetozone cf. zetlandica and *Chaetozone* sp. 9 is 10% (Table 2). COI distances with other species in the area vary between 19% and 26% (Table 2).

#### Distribution.

Norwegian coast and shelf, ~ 5–40 m depth.

### Chaetozone sp. 2 and sp. 4

Taxon classificationAnimaliaTerebellidaCirratulidae

4C9F4192-13CB-550E-9553-3850B16DA430

Figures 14

Chaetozone sp. 2 Grosse et al. 2020: fig. 4.
Chaetozone sp. 4 Grosse et al. 2020: fig. 4.

#### Material examined.

*Chaetozone* sp. 2: Norwegian Sea • 1 ind.; 61.42736°N, 7.47479°E; 18 Nov. 2012; 332 m; ZMBN125800 • 1; 62.48183°N, 4.46550°E; 10 Mar. 2012; 211 m; ZMBN125823 • 1 ind.; 61.0501°N, 5.40055°E; 03 May 2017; 1236 m; ZMBN117820 • 1 ind.; 62.482°N, 4.466°E; 03 Oct. 2012; 213 m ZMBN94537 • 1 ind.; 61.11299°N, 5.14124°E; 22 Jul. 2012; 354 m; NTNU-VM74503. *Chaetozone* sp. 4: Norwegian Sea • 3 ind.; 61.42736°N, 7.47479°E; 18 Nov. 2012; 332 m; ZMBN125796–125798 • 1 ind.; 61.21307°N, 5.03809°E; 14 Jul. 2015; 379 m; ZMBN125776 • 1 ind. ; 64.804°N, 10.111°E; 08 Oct. 2013; 378 m; ZMBN94483 • 1 ind.; 62.06827°N, 5.03811°E; 20 Jul. 2012; 334 m; ZMBN125781 • 2 ind.; 61.11299°N, 5.14124°E; 22 Jul. 2012; 360 m; NTNU-VM74500, 74507.

#### Comparative material.

*Chaetozone jubata*: Paratypes: Faroe-Shetland channel • 2 ind.; 61.5.57°N, 2.4093°W; Jul. 1996; 710 m; NMSZ.1999.237.4–5.

#### Diagnosis.

**Figure 14.**
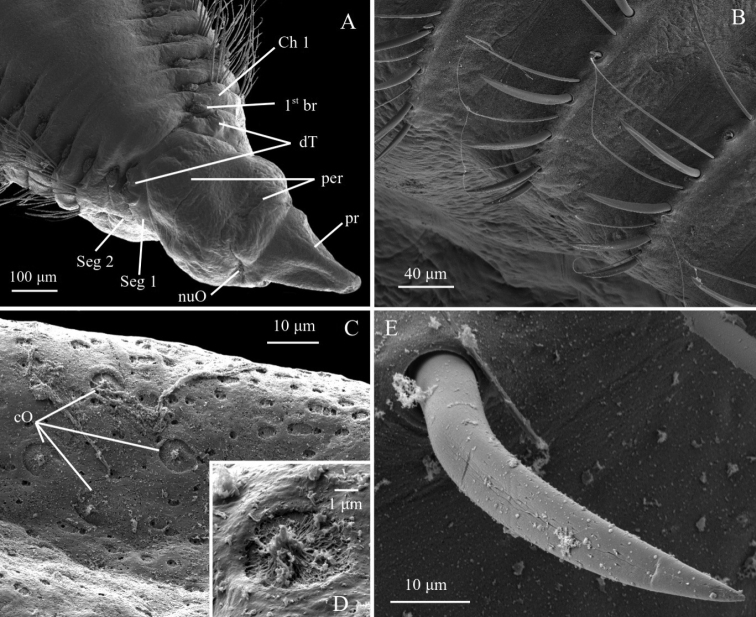
*Chaetozone* sp. 4. **A** ZMBN125798, SEM of anterior end in dorsal view **B** ZMBN125776, SEM of posterior spines **C** ZMBN125798, SEM of peristomium epidermis **D** ZMBN125798, SEM of ciliated organ **E** ZMBN125776, SEM of neuropodial spine. Abbreviations: br, branchiae; Ch, chaetiger; cO, ciliated organ; dT, dorsal tentacles; nuO, nuchal organ; per, peristomium; pr, prostomium; Seg, segment.

Prostomium fused with peristomium, giving the head a drop-like appearance; dorsal tentacles arising from segment 1 (achaetous), first pair of branchiae arising from segment 2 (achaetous); ventral groove; long capillary chaetae on expanded anterior, numerous long, broad flat spines on high complete cinctures (Fig. 14). No particular methylene blue staining pattern.

#### Molecular diagnosis.

*Chaetozone* sp. 2: COI: 130: G; 173: A; 202: T; 244: C; 293: C; 295: T; 309: G; 310: C; 395: G; 434: G; 454: T; 460: G; 470: G; 471: T; 477: C; 490: C; 302–303: AC; 564–565: CG. 28S: 408: A; 415: C; 416: A; 445: A; 571: C; 454–455: AG; 460–461: TC; 691–692: AA; 704–705: TC. (based on 8 COI sequences and 4 28S sequences) *Chaetozone* sp. 4: COI: 122: T; 487: G; 574: G (based on 10 COI sequences).

#### Remarks.

These considerably distinct molecular lineages but morphologically identical species, *Chaetozone* sp. 2 and *Chaetozone* sp. 4, are morphologically similar to *C. monteverdii* sp. nov. It is clear from the molecular analyses that they are different species from *C. monteverdii* sp. nov. Moreover, they are distinguished from *C. monteverdii* sp. nov. by having smooth long chaetae instead of segmented long chaetae, and a ventral groove instead of a ridge. Nevertheless, while none of the specimens of these two species examined present enlarged first segments, the condition present in *Chaetozone* spp. 2 and 4 could fit within the intra-specific variation documented in *C. monteverdii* sp. nov. The prostomium and peristomium in *Chaetozone* sp. 4 may also appear more distinctly separated than in the other species (Fig. 9). *Chaetozone* spp. 2 and 4 resemble *C. jubata*. However, they differ in the relative position of the paired tentacles and first pair of branchiae. It will be necessary to examine more specimens of each species, including of *C. jubata*, to investigate the intra-specific diversity in each group before confirming that neither *Chaetozone* sp. 2 nor *Chaetozone* sp. 4 are *C. jubata*.

The COI distance between *Chaetozone* sp. 2 and *Chaetozone* sp. 4 is 26% (Table 2). COI distances with other species in the area generally varies between 22% and 27%, with minimum at 9% between *Chaetozone* sp. 4 and *Chaetozone quinta* sp. nov. (Table 2).

#### Distribution.

Norwegian coast and shelf, offshore and in the fjords, where they occupy a wide range of depths from ~ 200 m to 1200 m depth.

## Discussion

While molecular studies are of major importance in species discovery, they too rarely lead to formal species descriptions (Pante et al. 2015). This is particularly the case when dealing with cryptic species: distinct species understood as separately evolving metapopulation lineages but morphologically identical (Fišer et al. 2018). However, even though they may not be morphologically diagnosable within a species complex, these species should be named and described. A way to do this is to provide molecular diagnostic characters along the morphological diagnosis (e.g., Churchill et al. 2014; Delić et al. 2017; Parapar et al. 2020; Teixeira et al. 2020). In a recent paper, analyses of DNA sequence data delimited a number of *Chaetozone* lineages compatible with the concept of species (understood as separately evolving metapopulation lineages (De Quieroz 2007)) (Grosse at al. 2020). In the present study, five of these are described as new, two are assigned to known species, and two are documented but not formally described. The diagnoses include both morphological and molecular characteristics.

Software currently available allow us to retrieve two types of molecular diagnostic characters: single bases or pairs of bases that together are diagnostic even if they may not be individually. While the software DeSignate (Hütter et al. 2020) makes it possible to create these pairs, or combined characters, by combining bases from any position in the alignment, we chose to include only pairs made by adjacent bases in species diagnosis. One reason is that it simplifies later usage. Another reason is that when allowing any distance within the bases, as many as 344 pairs were found in COI for *Chaetozone pseudosetosa* sp. nov. While molecular diagnostics typically include a few up to a couple of hundred single positions (e.g., Johnson et al. 2015; Teixeira et al. 2020), and all are potentially relevant, such a number is not manageable when describing a species or identifying it.

To be considered diagnostic, the same character state must be present for all specimens of a species, and absent from all other species (Davis and Nixon 1992; DeSalle et al. 2005). This leads to two considerations: the number of species involved in the analyses for comparison (reference group), and the number of sequences from the target species (query group). The more species (and the more sequences per species) there are in the reference group, the more chances are that at a given position in the alignment, at least one species possesses the same character state as the query group. This in turn means decreasing the chances of finding diagnostic characters, especially single bases. However, there is a trade-off, as with an important reference group, the diagnostic characters found in the query group are also more reliable, as we have more confidence in their uniqueness. Here we chose to use the complete *Chaetozone* COI dataset, including species not present in the North Atlantic and not discussed in this paper. The more sequences there are in the query group, the better overview we have of the intra-specific diversity. This once again means that there are less chances of finding diagnostic characters, but that the characters found will be more reliable. There have been several approaches to this issue, from describing species using molecular diagnostic characters based on a single individual and sequence (Jörger and Schrödl 2013), to limiting analyses and decisions to species with more than three sequences (Teixeira et al. 2020) and to pleading for wide datasets (Dayrat 2005). Here, *Chaetozone setosa* and *Chaetozone pseudosetosa* sp. nov. are the species for which there are most sequences, and have very few molecular diagnostic characters, but these should prove reliable. Although only two COI sequences were available from *Chaetozone quinta* sp. nov., we still included molecular diagnostic characters. However, some of these characters may not be diagnostic anymore when more specimens are sequenced, and more intra-specific diversity is known.

Another obstacle to the description of cryptic diversity within a species complex is the lack of sequenced material from type specimens or from specimens from type localities (see also Oug et al. 2014; Grosse et al. 2020), including that of other possible old and synonymised names, which is the first step to be able to assign existing taxon names to species, as it would allow comparing new sequences with these. The best procedure would be to sequence type material, but this is generally not possible as types are in most cases old specimens curated in museum collections, often fixed in formaldehyde, and with degraded DNA. *Chaetozone setosa*, and *C. pseudosetosa* sp. nov. are true cryptic species fitting the description of *C. setosa* but having distinct lineages (8% average p-distance in COI). Only the fact that recent material was available from the type locality of *C. setosa* made it possible to use this existing name for one lineage and give a new name to the other. However, several new species remain undescribed because there are several candidates for existing names. This is the case of Chaetozone cf. zetlandica in this study. In addition to this species, two other lineages are known from DNA analyses (approximately 10% average p-distance in COI) that could fit the description of *C. zetlandica*. However, *C. zetlandica* was described from Scotland, at a location from where no material is available for DNA sequencing at the moment.

Future addition of new specimens will contribute to knowledge of species groups, their geographical distribution, and possibly elucidate their morphological characteristics. In the meantime, molecular diagnostics can be of great help, in particular to identify cryptic species. Being among the most common and abundant groups of annelids in marine benthic environmental monitoring, knowledge of cirratulids and especially *Chaetozone* represents a step forward in understanding marine biodiversity.

Detailed knowledge of genetic diversity aggregated in described and defined morphogroups, although presently not named, is an advancement in understanding marine biological diversity. Genetic diversity is a compositional level in structural and functional diversity (Cochrane et al. 2016), has impact and is applicable in environmental monitoring and baseline studies in, for example, conservation planning and management. Genetic, as well as morphological, taxonomic diversity is fundamental in applying environmental DNA techniques to environmental monitoring. Thus, an overview of defined genetic groups and described morphogroups is important.

## Supplementary Material

XML Treatment for Chaetozone

XML Treatment for Chaetozone setosa

XML Treatment for Chaetozone pseudosetosa

XML Treatment for Chaetozone quinta

XML Treatment for Chaetozone barentsensis

XML Treatment for Chaetozone monteverdii

XML Treatment for Chaetozone chambersae

XML Treatment for Chaetozone cf. zetlandica

XML Treatment for Chaetozone

## Supplementary materials

Supplementary material 1 Specimens data Data type Collection data, Accession numbers Explanation note For all specimens and sequences used in this study, this file gives collection data and GeneBank/BOLD accession numbers. https://binary.pensoft.net/file/546243This dataset is made available under the Open Database License (http://opendatacommons.org/licenses/odbl/1.0/). The Open Database License (ODbL) is a license agreement intended to allow users to freely share, modify, and use this Dataset while maintaining this same freedom for others, provided that the original source and author(s) are credited.Maël Grosse, María Capa, Torkild Bakken

Supplementary material 2 COI alignment Data type DNA sequences Explanation note Contains all the COI sequences used in this study. https://binary.pensoft.net/file/546244This dataset is made available under the Open Database License (http://opendatacommons.org/licenses/odbl/1.0/). The Open Database License (ODbL) is a license agreement intended to allow users to freely share, modify, and use this Dataset while maintaining this same freedom for others, provided that the original source and author(s) are credited.Maël Grosse, María Capa, Torkild Bakken

Supplementary material 3 28S sequences Data type DNA sequences Explanation note Contains all 28S sequences used in this study. https://binary.pensoft.net/file/546245This dataset is made available under the Open Database License (http://opendatacommons.org/licenses/odbl/1.0/). The Open Database License (ODbL) is a license agreement intended to allow users to freely share, modify, and use this Dataset while maintaining this same freedom for others, provided that the original source and author(s) are credited.Maël Grosse, María Capa, Torkild Bakken
